# EXPECTATION AND ENGAGEMENT IMPACT COGNITIVE TRAINING BENEFITS IN MID-TO-LATE LIFE

**DOI:** 10.1093/geroni/igae098.0132

**Published:** 2024-12-31

**Authors:** Ashley Curtis, Amy Costa, Madison Musich, Anthony Schmiedeler, Kenda Eberhardt, Christina McCrae

**Affiliations:** University of South Florida, Tampa, Florida, United States; University of South Florida, Tampa, Florida, United States; University of Missouri, Columbia, Missouri, United States; University of Missouri, Columbia, Missouri, United States; University of Kansas Medical Center, Overland Park, Kansas, United States; University of South Florida, Tampa, Florida, United States

## Abstract

Lack of pharmacological agents for cognition has prompted research on behavioral treatments such as cognitive training (CT). Evidence regarding CT benefit is mixed. In accordance with the biopsychosocial model, we examined whether individual characteristics such as expectation for improvement and engagement levels impact CT benefits in middle-aged/older adults. In Study 1, older adults with insomnia (N=24) completed 6-week CT (Nintendo DS-Big Brain Academy; 60 mins/day, 3x/week) and waitlist control (WLC) in a cross-over design. At baseline and post-CT/post-WLC, participants completed NIH toolbox-cognitive tasks. In Study 2, middle-aged adults with generalized anxiety disorder (GAD; N=17) completed 8-week CT (Cognifit; 45 mins 3x/week). At baseline/post-CT, participants completed State-Trait Anxiety Inventory, Cognitive Failures Questionnaire, and computerized cognitive tasks. At mid-point, participants evaluated expectations of cognitive/anxiety improvement (0:none-100:extreme improvement). At post-treatment for both studies, participants completed the Game Engagement Questionnaire. Bivariate correlations evaluated associations between game engagement and improvement expectations on post-training proportional changes in cognitive (Study 1&2) and anxiety (Study 2) outcomes. In Study 1, greater perceived competence was associated with greater CT-related inhibition improvement (r=-.57, p=.008). In Study 2, greater expected level of cognitive improvement (r=-.58, p=.047) and sensory game engagement (r=-.730, p=.002) were associated with less CT-related working memory improvement. Greater CT perceived skill/competence was associated with more anxiety reduction (r=.59, p=.02). A biopsychosocial approach to intervention evaluation may be useful in CT trials in aging adults with insomnia and GAD. Psychological user characteristics such as expectation for improvement and perceived competence, as well as biological sensory engagement impact CT benefits.

